# On the Eau Medicinale d'Husson

**Published:** 1810-12

**Authors:** 


### *To the Editors of the Medical and Physical Journal.*

GENTLEMEN,

A CASE has been related in a recent publication, in which a violent vomitting and purging, accompanied with very great prostration of strength, and succeeded by convulsions, was produced by the celebrated *Eau Medicinale d'Husson.* In the same work, also, we are informed, that at Paris its use is prohibited by authority, in consequence of its violent effects, and the sudden death of several persons, in no long time after they had been cured of gout by this medicine.

Several cases of a similar nature are said to have happened in this country, and the deaths of two well-known gentlemen, either quite suddenly, or after a very short illness, have been attributed to this medicine; but, I am informed, there is no good foundation for this report; it is, however, of such a nature, that it ought either to be confirmed or refuted by those who are acquainted with the facts. In your last number, p. 364, you have inserted a case on the authority of a very respectable medical practitioner, which sufficiently demonstrates the alarming and dangerous effects of this nostrum under some circumstances, and it ought to warn all persons not to be too free in recommending a medicine, the composition of which is unknown, but which evidently consists of the most powerful and active ingredients.

The

The question has been frequently agitated, what ought to be the conduct of the regular Physician, with regard to the exhibition of secret or *quack* medicines? Should he sanction their use occasionally, or always avow himself the enemy of concealed remedies, and trust solely to his own skill and to approved and established formulas? Perhaps this question can only be decided by individual practitioners according to circumstances; if a patient earnestly wishes to try the efficacy of some of those secret remedies which are daily puffed off in the newspapers, and other vehicles of intelligence, by interested, duped, or duping persons, it may be sometimes prudent for his regular medical friend to comply with his wishes; but I am very much inclined to believe that the physicians of the present day* too readily accede to their patients' whims in this respect, and that they do not sufficiently consult their own dignity and consequence on such occasions.

We are not however to wonder, that so many medical men have employed the Eau Medicinale d'Husson, in their practice, recommended as it was by good authority, and capable as it proved of producing evident and decisive relief in paroxysms of the gout; but it is to be regretted, that after so much experienec has been had of it, so little, to be depended upon, is known of its effects. Of its utility in some cases of gout, no doubt can be entertained, but there is abundant reason to believe that so potent a remedy so indiscriminately given, must frequently have produced effects unexpected and unpromising. These ought to be known, in order that its real virtues and efficacy may be duly understood. Is it possible that any precise and clear knowledge could be gained of the most proper method of employing the bark, mercury, opium, digitalis, and other powerful articles of the Materia Medica, if their ill effects had not been freely commented on, as well as their good qualities pointed out? And is it not desirable that the good and bad qualities of the Eau Medicinale (and I will add of all other *quack* medicines†) should likewise be made public?

I should hope, gentlemen, that you will be supplied with a large body of evidence upon the subject of the Eau Medicinale; to insert in your valuable pages, *all* the cases that might be trans-

---

* The principal Physicians of London, in the days of Doctor James, refused to prescribe his powder, because the composition of it was unknown to them; and whenever he was called in, they retired from their attendance. Yet Dr. James was a regular bred Physician, and a man of considerable learning and judgment.

† I believe nothing would tend more to discountenance quack medicines, than such an investigation of their good and ill effects, carefully conducted, and fairly laid before the public. By this means the very few useful nostrums would be known and properly employed, and the noxious and effete ones (by far the greater number) would be consigned to the neglect they deserve.

mitted mitted to you of the good and ill effects of this mysterious nostrum, could not perhaps answer your purpose or be well received by your readers, but I think that the result of the experience of many practitioners might by your means be collected, and be candidly given to the public. That great advantages would be derived from such an exposition of facts is most certain, the Eau Medicinale would then soon settle at its proper level; it would be carefully and cautiously prescribed by practitioners of skill and judgment, who would probably improve its good qualities, and correct what was found to be noxious and prejudicial. At present it seems to be given in a most loose and irregular manner, without any consideration either of the patient's age, constitution, or manner of life; it is given indiscriminately either at the beginning, the middle, or the end of a fit of the gout; nobody seems to know what kind of operation is to be expected from it, or what regimen is to be observed during its use, yet every body acknowledges that its operation is sometimes alarmingly violent, and your pages shew that it is sometimes imminently dangerous.

I remain, &c.

*Nov.* 12, 1810. REPREHENSOR.

